# Cyclic Olefin Copolymer Interleaves for Thermally Mendable Carbon/Epoxy Laminates

**DOI:** 10.3390/molecules25225347

**Published:** 2020-11-16

**Authors:** Riccardo Costan Zovi, Haroon Mahmood, Andrea Dorigato, Giulia Fredi, Alessandro Pegoretti

**Affiliations:** 1 Department of Industrial Engineering, University of Trento, 38123 Trento, Italy; riccardo.costanzovi@alumni.unitn.it (R.C.Z.); andrea.dorigato@unitn.it (A.D.); giulia.fredi@unitn.it (G.F.); 2 National Interuniversity Consortium of Materials Science and Technology (INSTM), 50121 Florence, Italy

**Keywords:** thermal mending, self-healing, cyclic olefin copolymer, laminates, carbon fiber, epoxy

## Abstract

Thin cyclic olefin copolymer (COC) foils were used as intrinsic thermoplastic healing agents in carbon fiber (CF)-reinforced epoxy laminates. COC films were produced by hot pressing and were interleaved in the interlaminar regions between each EP/CF lamina, during the hand layup fabrication of the laminates. Three samples were produced, i.e., the neat EP/CF laminate without COC, and two laminates containing COC layers with a thickness of 44 μm and 77 μm, respectively. It was observed that the fiber volume fraction decreased, and the porosity increased with the introduction of COC layers, and this effect was more evident when thick films were used. These two effects, combined with the sub-optimal adhesion between COC and EP, caused a decrease in the mechanical properties (i.e., the elastic modulus, flexural strength, interlaminar shear strength and interlaminar fracture toughness) of the laminates. Specimens subjected to mode I interlaminar fracture toughness test were then thermally mended under pressure by resistive heating, through the Joule effect of conductive CFs. A temperature of approximately 190 °C was reached during the healing treatment. The healing efficiency was evaluated as the ratio of critical strain energy release rate (*G_IC_*) of the healed and virgin specimens. Healed specimens containing COC layers of 44 μm and 77 μm exhibited a healing efficiency of 164% and 100%, respectively. As expected, the healing treatment was not beneficial for the neat EP/CF laminate without COC, which experienced a healing efficiency of only 2%. This result proved the efficacy of COC layers as a healing agent for EP/CF laminates, and the effectiveness of resistive heating as a way to activate the intrinsic healing mechanism.

## 1. Introduction

Carbon fiber reinforced polymers (CFRPs) are in use since the 1950s in the aerospace industry, to fill the need for materials with better specific properties, compared to those of metals [1,2]. In fact, structural polymer composites are of interest especially in the transportation field, as they lead to the production of lightweight components with great structural performance, which allow saving fuel and decreasing CO_2_ emissions [3]. These materials are also attractive for the possibility of pairing the mechanical function with additional functionalities, such as strain monitoring, self-healing and energy storage capabilities [4,5,6,7,8].

Typical failure mechanism of thermosetting fiber-reinforced polymer composites (FRPCs) include interfacial delamination and matrix cracking [9,10,11]. These materials are also vulnerable to impact damage due to a lack of plastic deformation, which could lead to interlaminar cracks [12]. To mitigate this behavior, the design of composite structures is often pursued with a damage-tolerant approach. For example, CFRPs used in aerospace industry are typically designed with an allowable compressive strain level lower than 0.4%, whereas commercially available carbon fiber composites have a compressive strain to failure of approximately 1% [13]. Such conservative design produces overweight structures that reduce the advantages of high strength-to-weight and stiffness-to-weight ratios of composite structures.

The repairing methods of such composites with a thermosetting matrix comprise either the replacement of the entire component or the injection of new material in the damaged part, which are expensive processes requiring skilled manual intervention [10,14]. Moreover, the first stage of damage nucleation entails cracks located in the matrix, which can be difficult to detect and repair, due to their limited dimensions [15]. In addition, microcracking is one of the most severe damages generated in service, which can lead to the failure of the material and shorten the lifetime of the structure [16]. These phenomena are at the basis of the interest towards composite materials with self-healing abilities, grown since the 1990s and inspired by the response of natural and biological materials to damage [17,18]. To obtain such healing response in man-made materials, it is necessary that atoms or molecules flow from their initial position to the damaged zone and restore the physical contact between both crack faces [19,20]. This could be achieved either by extrinsic or by intrinsic self-healing mechanisms.

In the extrinsic (or autonomous) mechanism, healing is accomplished by including a liquid healing agent and a liquid catalyst in brittle vessels such as microcapsules, hollow fibers, or microvascular systems [10,21]. Such brittle vessels are broken by a crack propagating in the matrix, with the consequent release of the healing agent and the catalyst, which migrate to the crack faces through capillary forces and react with each other, thereby closing the crack [10,16]. The healing efficiency obtained with this method, defined as the mechanical strength ratio between the healed system and the virgin system, was close to 100% [10]. However, this healing action can be performed only once in each region, due to the thermosetting nature of the involved materials. On the other hand, the intrinsic (or non-autonomous) self-healing mechanism is based on reversible physical and chemical interactions, which are governed by molecular mobility, through the application of external heat or pressure [21].

Intrinsic self-healing can be achieved by modifying the thermosetting matrix, by dispersing a thermoplastic polymer phase. Whenever a crack is formed, the component can be heated above the softening temperature of the thermoplastic polymer, which can eventually migrate to the crack zone and fill it. Although this mechanism is non-autonomous and needs an external stimulus, it still possesses some advantages as compared to extrinsic systems, as it does not require complicated encapsulation processes of reactive systems. Moreover, this healing mechanism can be repeatedly performed various times in the same zone [16].

Intrinsic self-healing of thermosetting composites is realized by using several types of thermoplastic polymers. Karger-Kocsis [22] studied the self-healing effectiveness of different types of epoxy resins (EP) filled with various amounts of poly(ε-caprolactone) (PCL). The author reported that the dispersion of the PCL phase in the EP matrix depended on the EP type, and the study revealed a healing efficiency of up to 80%, with a healing temperature of 80 °C. In 2009, Luo et al. [23] studied similar EP/PCL blends and were able to reach a healing efficiency above 100%, with a healing temperature of 190 °C. Inspired by this works, our group in 2020 for the first time investigated the thermal mending potential of a cyclic olefin copolymer (COC) in an EP matrix [21]. COC is an attractive class of amorphous thermoplastic polymers, thanks to their high transparency, good heat resistance, low moisture absorption, good chemical resistance, low density, and elevated stiffness [12,24,25,26,27,28]. Several EP/COC blends were prepared by mechanically mixing the COC powder and EP resin in the uncured state. The cured samples were broken and healed under a compressive stress of 15 MPa at 190 °C for 1 h, and the resulting healing efficiency was close to 100% for the sample with a COC content of 40 wt.%. 

Due to the high healing efficiencies, thermosetting polymers containing a dispersed thermoplastic phase also attracted great attention as matrices in fiber-reinforced composites [29,30,31,32,33]. However, up to now, the healing agent was mainly added to the laminates in form of fine particles. One drawback associated with such systems is the significant increase of the viscosity of the blend (particulate or immiscible blend). Such situation can represent a limitation in the fabrication of composites by resin infusion processes. One possibility is to insert the healing agent as an interlayer, prior to the infusion of resin. Various efforts were made in the past to investigate the feasibility of using an interlayer in laminate for self-healing purposes like complex coaxial electrospun mats [34,35], films [36,37], polymer mesh [38], etc. Such approach simplifies the laminate fabrication process, i.e., without any modification of resin, semi-automated process like resin transfer molding becomes feasible. Resultantly, the produced laminates would not contain production defects like voids, incomplete fiber infusion, etc.

There are different possible ways to provide heat to the system in order to activate the self-healing process, among which, the most commonly used is the thermal treatment of the composite under pressure in an autoclave. Another interesting solution that can be exploited with electrically conductive fibers is the use of resistive heat evolved by the Joule heating effect when an electrical current is passed through a material. In CFRPs, the fibers can be treated as resistive elements, while the surrounding matrix acts as an insulator. For this reason, almost the totality of the electrical conduction occurs in the fibers [39]. The effectiveness of healing of a composite material by resistive heating was demonstrated by Park et al. [40] in 2008. They induced microcracks by three-point bending on the samples and then they healed these samples through resistive heating, by applying the electrical contacts directly to the specimen surfaces. The specimens were effectively repaired after the healing process, performed in the temperature range of 70–100 °C for some minutes.

On the basis of these considerations, the present study investigates the healing potential of thin COC foils in epoxy/carbon fiber (EP/CF) laminates. The first novelty of this work is the use of COC not as particles dispersed in the matrix mixture, but as thin films produced by compression molding, and included in the interlaminar region of the laminates. The second novelty is the Joule heating, performed via a new lab-made device capable of applying an external current and pressure simultaneously. The aim of this work was, therefore, to evaluate for the first time the thermal mending potential of COC thin films of two different thicknesses (44 μm and 77 μm) inserted into the interlaminar region of EP/CF laminates. The laminates were subjected to an in-depth microstructural and thermomechanical characterization, and the thermal mending capability of COC was evaluated by comparing the fracture toughness of virgin and healed laminates.

## 2. Materials and Methods

### 2.1. Materials

A bi-component epoxy system used as a thermosetting polymer matrix was provided by Elantas Europe S.r.l. (Collecchio, Italy). It was composed of an epoxy resin (Elan-tech EC 157.1) and an aminic hardener (Elan-tech W342). The selected reinforcement was a hybrid unidirectional fabric (GV-201 U TFX) provided by Angeloni s.r.l. (Quarto d’Altino, VE, Italy), consisting of high strength CF fabric (200 g/m^2^) and thermoplastic-coated glass yarns (weft, 17 g/m^2^). The COC pellets of TOPAS^®^ 8007, supplied by TOPAS Advanced Polymers GmbH (Kelsterbach, Germany), were used to produce COC thin films. According to the producer’s datasheet, this COC consisted of 65 wt.% ethylene and 35 wt.% norbornene and had a density of 1.02 g/cm^3^, a glass transition temperature (T_g_) of 78 °C, and a melt flow index (MFI) of 1.7 g/10 min (190 °C, 2.16 kg).

### 2.2. Sample Preparation

#### 2.2.1. COC Thin Films

COC thin films were produced by using a Carver 4122E hot press (Carver, Inc., Wabash, IN, USA). COC pellets were disposed with a precise interparticle distance between two copper plates, covered by polyethylene terephthalate (Mylar^®^) sheets. The pellets were positioned with a lab-made wooden stencil with 98 holes placed at a distance of 2.0 ± 0.2 cm, in the longitudinal and transversal direction. This distance was calculated based on how much a single COC pellet spread on the surface, under a pressure of 4.1 MPa at a temperature of 190 °C. The wooden stencil was removed before compression molding. This system was used to produce films with two different thicknesses, namely 44 μm (44COC) and 77 μm (77COC). To produce 44COC films, one COC pellet was added into each hole of the stencil (98 grains), while for the 77COC, two pellets were added per hole (196 grains). This production process guaranteed the production of thin films with uniform thickness. Both film types were produced at 190 °C under an applied pressure of 4.10 MPa for 44COC and 2.74 MPa for 77COC, respectively. The pellets were pre-heated in the press for 1 min and then the pressure was applied for 14 min.

#### 2.2.2. EP/COC/CF Composites

**Figure 1:**
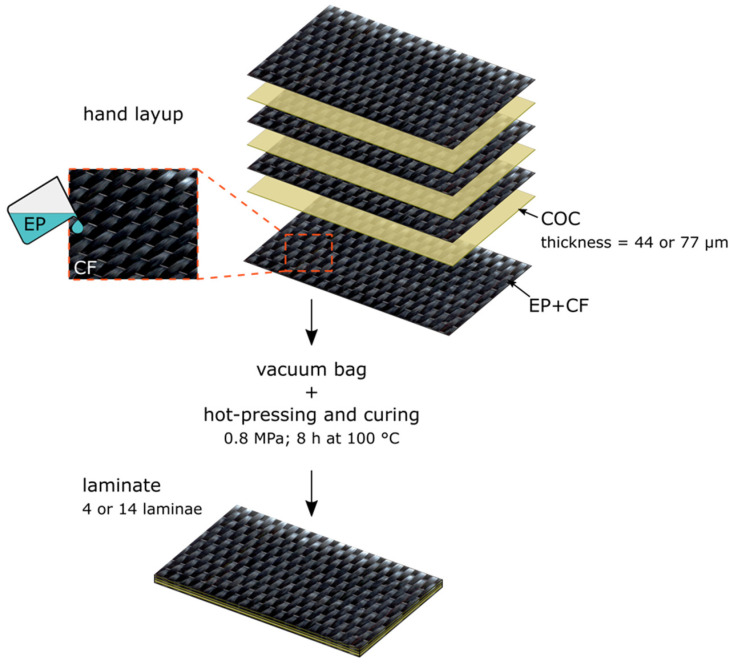
Schematic diagram representing the fabrication process of EP/COC/CF hybrid composite.

For the preparation of the unidirectional EP/COC/CF composites, first the epoxy base and the hardener were mixed at a relative weight ratio of 100:30. This mixture was used as matrix to prepare laminates with the carbon fiber fabric and the COC films, via hand-layup and vacuum bagging techniques. Two different hybrid composites were prepared—the first containing COC films with a thickness of 44 μm (EP/44COC/CF) in each interlaminar region, and the second with COC films with thickness of 77 μm (EP/77COC/CF). Each film was placed between two layers of CF in such a way that a thin layer of epoxy matrix was always between the COC film and CF fabric. A neat EP/CF composite without COC was produced as a control sample. Figure 1 shows a schematic diagram of the fabrication of these unidirectional EP/COC/CF hybrid composites.

Each laminate type was produced with two different numbers of laminae. Thick composites with 14 CF laminae were produced to test the interlaminar fracture toughness and interlaminar shear strength. A thin PET film (26 μm) was inserted in the mid-plane, in order to create a pre-crack to be propagated under controlled conditions during the interlaminar fracture toughness test. Additionally, thin composites with 4 CF laminae were fabricated to produce specimens for three-point bending and electrical resistivity tests.

The prepared laminates were vacuum bagged for 15 min, with a vacuum pump (Edwards RV5) and then they were put in a compression molding apparatus (Carver 2699) and cured for 8 h at 100 °C, under a pressure of 0.8 MPa. This curing cycle, chosen among those indicated by the resin manufacturer, allowed to crosslink the matrix at a temperature above the glass transition temperature (T_g_) of COC, in order to promote the adhesion between EP and COC. The prepared laminates were weighed to determine the weight distribution of the components. The nomenclature, composition, and thickness of the prepared laminates is reported in Table 1.

### 2.3. Characterization Techniques

The microstructure of the EP/CF and EP/COC/CF composites was analyzed with a Zeiss Axiophot optical microscope (Carl Zeiss AG, Oberkochen, Germany), coupled with a Leica DC300 digital camera (Leica Microsystems Ltd., Heerbrugg, Switzerland). In the case of the EP/COC/CF laminates, both the unhealed (virgin) and healed samples were analyzed. The transversal and longitudinal sample cross-sections were embedded in an epoxy resin and cured for 24 h at room temperature. Then, the samples surface was polished by abrasive grinding papers made of silicon carbide with grit polishing size of 240, 800, 1200, and 4000, sequentially. Finally, polishing was performed with cloths impregnated with 3 μm and 1 μm diamond particles.

The experimental density of the samples EP/CF and EP/COC/CF was measured with the liquid displacement method at 23 °C, by using a Mettler-Toledo ME104 (Schwerzenbach, Switzerland) precision balance, with a sensitivity of 10^−4^ g. Samples were weighted in air and in ethanol, following the Standard ASTM D792-13. The density of CF was measured with a Micromeritics^®^ Accupyc 1330 helium pycnometer (Micromeritics Instrument Corporation, Norcross, GA, USA) at 23 °C, by using a testing chamber of 3.5 cm^3^. Then, the theoretical density of the composites (*ρ_t_*) was evaluated with Equation (1):

(1) $$\rho_{t}=\frac{1}{\frac{\omega_{f}}{\rho_{f}}+\frac{\omega_{m}}{\rho_{m}}+\frac{\omega_{COC}}{\rho_{COC}}}$$

where $\omega_{f}$, $\omega_{m}$, and $\omega_{COC}$ are the weight fractions of carbon fibers, matrix (epoxy + hardener) and COC, respectively (see Table 1), and $\rho_{f}$, $\rho_{m}$, and $\rho_{COC}$ are the density of each of these phases, equal to 1.78 g/cm^3^, 1.15 g/cm^3^, and 1.01 g/cm^3^, respectively. The experimental density $\left(\rho_{exp}\right)$ of the prepared composites was then compared with the theoretical density, and the void volume fraction $\left(\vartheta_{v}\right)$ was calculated, as described in Equation (2):

(2) $$\vartheta_{v}=\frac{\rho_{t}-\rho_{exp}}{\rho_{t}}$$

Thermogravimetric analyses (TGA) were performed through a Mettler TG50 (Mettler-Toledo GmbH, Schwerzenbach, Switzerland) machine, in order to investigate the thermal stability of unhealed EP/CF, EP/44COC/CF, and EP/77COC/CF samples. The tests were carried out from 25 °C to 700 °C, at a heating rate of 10 °C/min and under a constant nitrogen flow of 100 mL/min. This test allowed the calculation of $T_{onset}$, i.e., the starting degradation temperature calculated as the intersection of the tangents of the curve, before and after the start of the degradation, $T_{d\_EP}$ and $T_{d\_COC}$, i.e., the peak temperatures of the mass loss derivative signal at the degradation of the EP and COC phases, respectively, and $m_{r}$, i.e., the residual mass at 700 °C, from which the weight percent of CF present in the composite was determined.

Differential scanning calorimetry (DSC) analysis were performed through Mettler DSC30 calorimeter (Mettler-Toledo GmbH, Schwerzenbach, Switzerland) to investigate the thermal behavior of the unhealed EP/CF, EP/44COC/CF, and EP/77COC/CF samples. The analysis was performed in nitrogen atmosphere with a constant flow of 100 mL/min, and three scanning steps were performed, i.e., a first heating phase from 0 °C to 130 °C, a cooling phase from 130 °C to 0 °C, and a second heating phase from 0 °C to 130 °C. A heating/cooling rate of 10 °C/min was adopted. The test allowed the determination of the T_g_ of epoxy and COC.

Flexural properties of the unhealed EP/CF, EP/44COC/CF, and EP/77COC/CF samples were evaluated by using an Instron^®^ 5969 universal testing machine (Norwood, MA, USA). According to the ASTM D790-15 standard, the rectangular samples were produced from the 4-layer laminates, with a cross-section with the nominal dimensions of 12 × 1 mm^2^. A span to depth ratio of 60:1 was imposed for the flexural modulus measurements, while a ratio of 40:1 was fixed for the flexural strength evaluation. According to the standard, a cross-head speed was imposed to obtain a strain rate of 0.01 mm^−1^ on the outer surface of the samples. The flexural modulus, strength, and strain at break were evaluated, as described in the ASTM D790 15 standard. At least five specimens were tested for each composition.

Short beam shear (SBS) test was performed to evaluate the interlaminar shear strength (ILSS) of the EP/CF and EP/COC/CF samples, according to the ASTM D2344 standard. Fourteen-layer composites were tested under three-point bending configuration, with an Instron^®^ 5969 universal testing machine. Specimens for three-point bending tests were prepared with a length equal to six times the thickness and the width equal to two times the thickness. The tests were performed by imposing a cross head speed of 1 mm/min. The adopted support span length was four times the thickness of the specimen. The test was terminated when a load drop of 30% was reached or the cross-head displaced more than the thickness of the specimen. At least five specimens were tested for each composition. The *ILSS* was evaluated using the Equation (3):

(3) $$ILSS=0.75\times \frac{P_{m}}{b\times h}$$

where *P_m_* is the maximum load, and *b* and *h* are the width and thickness of the specimen, respectively.

**Figure 2:**
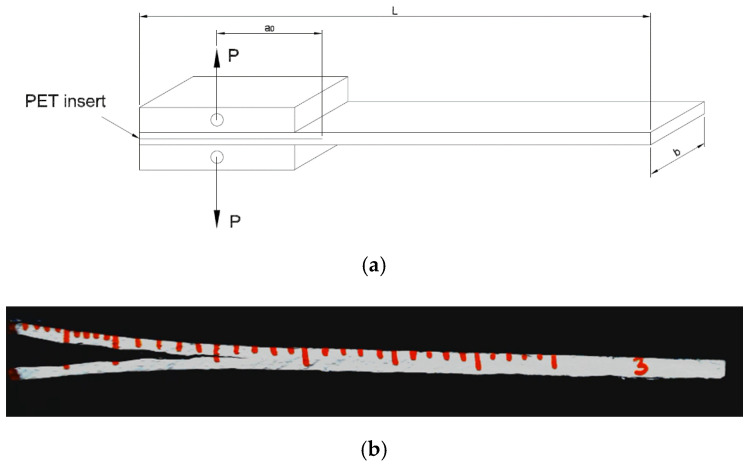
Mode I interlaminar fracture toughness sample. (**a**) Dimensions and configuration, and (**b**) example of crack propagation during a test.

In order to evaluate the fracture toughness of the EP/CF and hybrid EP/COC/CF laminates, an interlaminar fracture toughness test was performed, according to ASTM D5528-13. These tests were conducted using an Instron^®^ 5969 universal testing machine with a cross-head speed of 2.5 mm/min, on double cantilever beam (DCB) samples with nominal dimensions of 150 × 23 × 3 mm^3^. Two loading blocks were bonded to the specimen at 50 mm, far from the starting crack tip $\left(a_{0}\right)$. The specimen configuration is reported in Figure 2a. To record the crack length during the test, a 60 mm graduated scale with 1 mm accuracy was drawn on the lateral side of each sample, as shown in Figure 2b.

The crack advancement was monitored with a digital webcam (Logitech^®^ B910HD). The specimens were pre-cracked by loading them until 5 mm of crack advancement, followed by unloading and reloading for fracture toughness testing, until a crack advancement of 50 mm. During the tests, the applied load (P), crack opening displacement (δ) and crack length (a) values were measured, and the mode I interlaminar fracture toughness $\left(G_{I}\right)$ was calculated via Equation (4):

(4) $$G_{I}=\frac{3P\delta }{2b\left(a+\left|\Delta \right|\right)}$$

where *b* is the specimen width and |Δ| is a factor used to correct the vertical displacement and rotation effects at the delamination crack tip. In this way, the interlaminar fracture toughness $\left(G_{IC\_v}\right)$ of the virgin EP/CF, EP/44COC/CF, and EP/77COC/CF samples was evaluated. After the test, the samples were repaired, as described in Section 2.4.2, and the same tests were performed again to calculate the healing efficiency. To evaluate the healing efficiency of the laminates, the initiation values of G_IC_ were selected on the basis of the point where delamination was visually observed (VIS). This allowed the determination of the mode I interlaminar fracture toughness of the virgin ($G_{IC\_v}^{VIS}$) and repaired $\left(G_{IC\_r}^{VIS}\right)$ specimens, corresponding to VIS. The apparent healing efficiency $\left(\eta_{VIS}\right)$ of the prepared samples was thus computed, as reported in Equation (5):

(5) $$\eta_{VIS}=\frac{G_{IC\_r}^{VIS}}{G_{IC\_v}^{VIS}}\times 100$$

Finally, the microstructural features of the healed EP/CF and EP/COC/CF composites were analyzed by using a Zeiss Axiophot optical microscope (Oberkochen, Germany), coupled with a Leica DC300 digital camera (Leica Microsystems Ltd., Heerbrugg, Switzerland).

### 2.4. Healing of the Composite Samples

#### 2.4.1. Electrical Resistivity Measurements

Resistivity measurements were performed on the laminates with a four-probe method, in order to evaluate the possibility of heating the sample through Joule effect, by applying a constant voltage. From 4-layer EP/CF and EP/COC/CF laminates, rectangular specimens were produced with nominal dimensions of 40 × 13 × 1 mm^3^. At least five samples for each composition were tested. Silver-based paint was put on the surfaces in contact with the voltmeter electrical terminals, and on the lateral sides of each specimen, to improve the electrical conductivity of the surfaces [41]. The good quality of the contacts is crucial to produce reliable and comparable results. A DC electricity generator (ISO-TECH IPS 303DD) and two digital multimeters (ISO-TECH IDM 67) were utilized. By settling an output voltage of the electrical generator at 0.1, 0.2, 0.3, 0.5 V, the current and the voltage passing through the samples were recorded. The distance between the voltage contacts $\left(l^{\prime }\right)$ was set at 11 mm. The test was performed with a lab-made device, composed of two aluminum electrodes, one fixed and one movable transversely, covered with two copper plates. A compressive force to the mobile electrode was applied by means of an apparatus that transforms an applied torque into a translational force. This was done not only to hold the sample during the test but also to ensure that the same compression force was always applied on all specimens, which is important because the electrical resistivity is greatly affected by the applied pressure. In fact, a compression stress in the through-thickness direction produces a decrease in the electrical resistivity [42], due to an increase of the fiber orientation. On the contrary, a compression stress in the fiber direction produces an increase of the electrical resistivity [43]. The current was applied on the specimens by means of electrical terminals connected to the copper electrode of the device, while the voltage was measured by electrical terminals connected directly on the specimen surfaces. The four-point resistivity of the samples was evaluated using Equation (6):

(6) $$\rho =\frac{Vwh}{Il^{\prime }}$$

where ρ is the resistivity (Ω.mm), V is the voltage (V), w is the width of the sample (mm), h is the thickness of the sample (mm), I is the current the ammeter measures flowing through the sample (A), and $l^{\prime }$ is the distance between the two points where the voltmeter wires make contact with the sample (mm).

#### 2.4.2. Self-Healing by Joule Heating Mechanism

**Figure 3:**
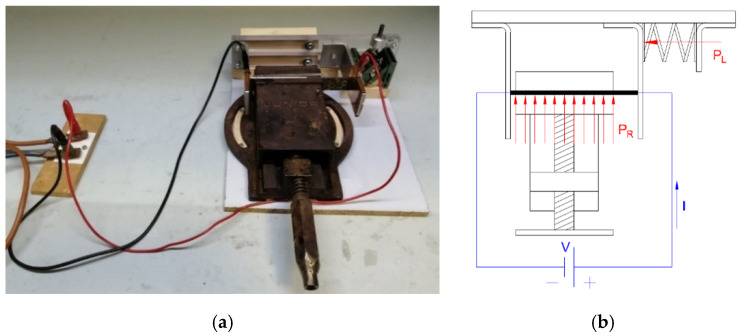
(**a**) Lab-made Joule heating repairing device, and (**b**) schematic drawing of the Joule heating repairing mechanism (top view).

The healing of the delaminated samples was performed by exploiting the Joule heating effect through a lab-made device. The device was composed of two steel electrodes covered with copper plates, in order to enhance their electrical conductivity. Here, the current was applied through electrical terminals (Figure 3). The lateral surfaces of the specimens were covered with silver paint to increase the electrical conductivity of the contacts. Similar to resistivity measurements, one of the electrodes was fixed, while the second was free to move in translational direction, through the application of a force. The pressure $\left(P_{L}\right)$ applied to the specimens was equal to 180 kPa, which was the minimum pressure that the torque screw was able to apply. A layer of PET between each copper plate and steel was inserted to decrease the electrical losses through the steel supports, converging the current inside the tested specimen. To activate the intrinsic self-healing mechanism on the fracture surfaces, a repairing pressure $\left(P_{R}\right)$ of 500 kPa was applied, by using a torque screw vice. This pressure level was selected according to the indications obtained in a previous work on the self-healing behavior of epoxy/COC blends [44]. A piece of glass ceramic was inserted between each side of the vice shoulders and the specimen, to minimize heat losses through the steel vice. The specimen was separated from the glass ceramic by polytetrafluoroethylene (PTFE) sheets, to ease the detachment of the sample. The current was applied by using a direct current electricity generator (Agilent 6674A) that is able to apply a maximum voltage of 60 V and a maximum current of 35 A. The voltage and current parameters were manually set for each sample, to obtain a temperature on the crack surface in a range between 170 °C and 190 °C for 1 h. This healing temperature was selected according to the indications of our previous work on the self-healing behavior epoxy/COC blends [21,44].

**Figure 4:**
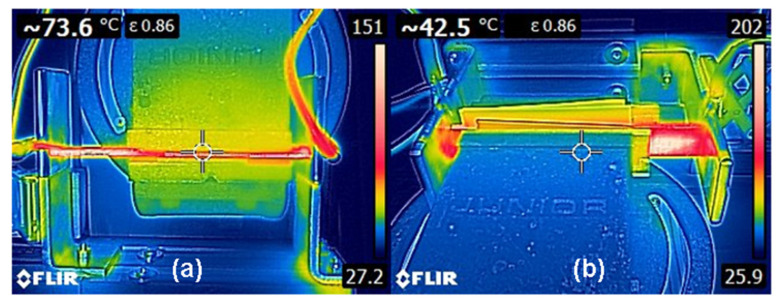
(**a**) Example of top view temperature profile of sample EP/44COC/CF, before application of thermal insulating material between the vice and the specimen. (**b**) Example of frontal view temperature profile of sample EP/44COC/CF, after application of the thermal insulating material between the vice and the specimen.

The temperature reached by the samples during the healing process was measured by using a thermocouple (RS 1319A K-Type Thermometer) and an infrared thermal imaging camera (FLIR E6). The thermocouple was used to check the temperature on the crack surfaces inside the sample, which was on average 10 °C higher than the surface temperature. By using the thermal imaging camera, the surface temperature and its profile was continuously checked during the repairing operation, to ensure that the temperature was always in the range 160–200 °C. Examples of temperature profiles obtained during the healing process on the EP/44COC/CF are reported in Figure 4. According to Figure 4a, the vice does not appear to be thermally insulated and the temperature reached on the crack external surface was not high enough. The temperature profile seems to be homogeneous and it is representative of the desired profiles obtained for all tested specimens. Due to the presence of a thermally insulating material between the vice and the specimen, the vice was well insulated, and the temperature profile was reasonably homogeneous (Figure 4b). The homogeneity of the temperature profile on the whole surface of the sample was dependent on the quality of the electrical contacts, between the lateral side of the specimen covered with silver paint and the electrodes.

## 3. Results and Discussion

### 3.1. Characterization of the Composites

**Figure 5:**
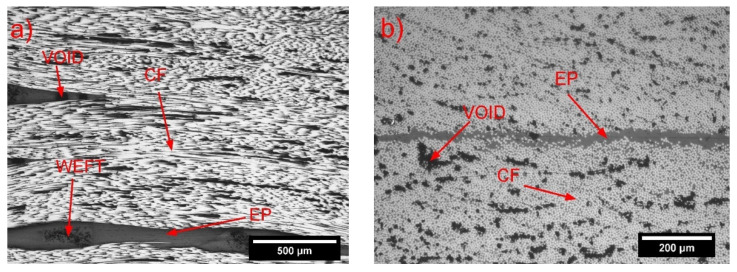

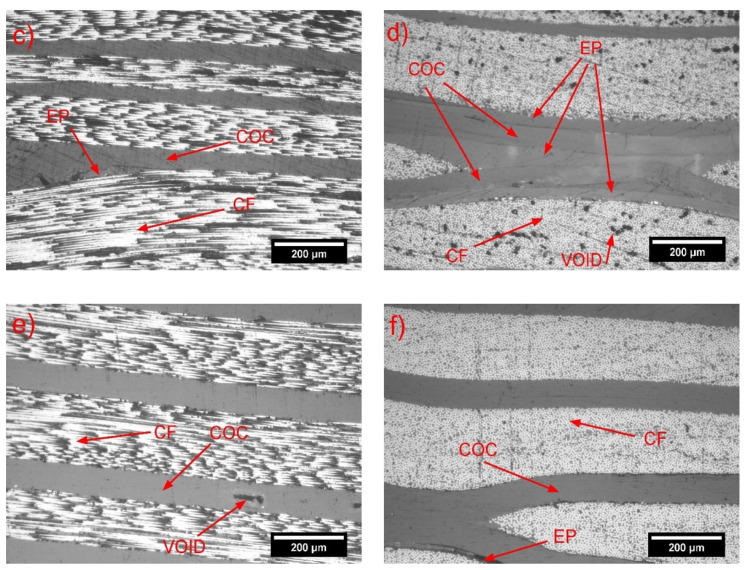
Optical microscope images of the polished surfaces of the unhealed specimens (4-layer samples): (**a**,**b**) EP/CF; (**c**,**d**) EP/44COC/CF; and (**e**,**f**) EP/77COC/CF. Longitudinal view (**a**,**c**,**e**) and cross-sectional view (**b**,**d**,**f**).

The optical microscope pictures of the longitudinal and cross-sectional view of the unhealed samples (4-layer laminates) are reported in Figure 5. The EP/CF samples (Figure 5a,b) present some matrix-rich zones, close to the weft of the fabric, which both act as defect in the material and negatively affect the mechanical properties of the composite. Figure 5c,d show the EP/44COC/CF samples, and the COC films could be observed between the CF layers. The COC layers were parallel to the fiber orientation, except for the areas close to the fabric weft. Between the COC layers, some matrix-rich areas were present.

The EP/CF layers were always separated by a COC film, meaning that the response of the delamination tests performed would probably be influenced by the adhesion between the COC film and the matrix. In the case of the EP/77COC/CF sample (Figure 5e,f), the presence of EP was rather limited between the CF and COC. This apparently lowers the amount of epoxy, compared to EP/44COC/CF, which was confirmed by the volumetric composition of the composites, reported in Table 2.

An increase in the thickness of the COC films caused a decrease in the CF volume fraction and an increase in the void volume fraction. The void content in the laminates containing COC was approximately twice that detected for the EP/CF laminate, as it reached a value of 6.4 vol% for the EP/77COC/CF composite, which could impair the ultimate tensile properties and the fracture toughness of the prepared laminates. This increment of porosity was partially due to the presence of a continuous impermeable film of COC, through which the air bubbles could not be efficiently removed by applying vacuum in the lateral (thickness) direction. This problem could be avoided in the future by fabricating laminates through resin infusion methods, which involve the impregnation of the laminate and the air removal, along the fiber direction.

**Figure 6:**
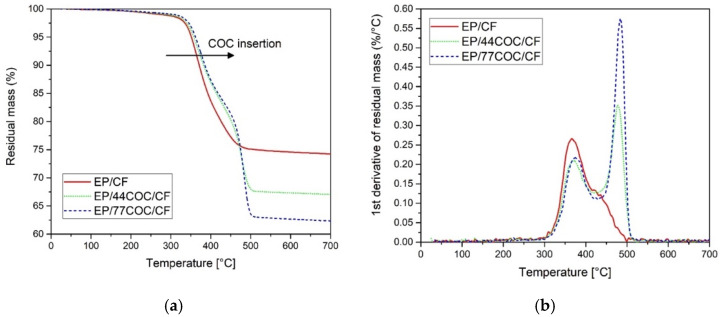
Results of the TGA tests on EP/CF, EP/44COC/CF and EP/77COC/CF laminates. (**a**) Residual mass and (**b**) derivative of the mass loss as a function of temperature.

TGA analysis was performed to investigate the thermal degradation resistance of the prepared composites. Figure 6 shows the trends of the residual mass and mass loss derivative, as a function of temperature, obtained from the 4-layer laminates. Table 3 reports the values of $T_{onset}$, $T_{d\_EP}$, $T_{d\_COC}$, and $m_{r}$ of these laminates.

The neat laminate EP/CF shows a single degradation step, associated with the degradation of EP phase, at approximately 366 °C. The same step was observable in the laminates containing COC, at a slightly higher temperature, and it was followed by the degradation step of the COC phase at approximately 480 °C. The initial degradation temperature of the composites $\left(T_{onset}\right)$ increased slightly with COC addition, passing from 336 °C of EP/CF to 342 °C and 344 °C for EP/44COC/CF and EP/77COC/CF, respectively. Due to a higher COC content and a consequent lower weight fraction of CF, the residual mass decreased by increasing the thickness of the COC layers.

**Figure 7:**
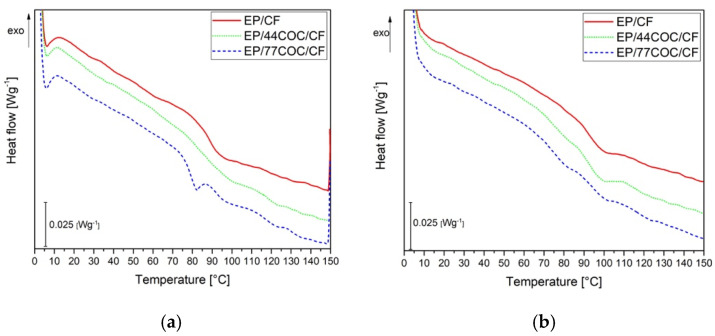
DSC thermograms on the prepared laminates. (**a**) First heating scan and (**b**) second heating scan.

DSC analysis was carried out to investigate the thermal transitions of COC and the influence of COC on the T_g_ of the EP phase. Figure 7 shows the DSC thermograms of the first and the second heating scans of the prepared laminates, while Table 4 reports the most important DSC results.

Even if $T_{g_{\_EP}}^{1}$ and $T_{g_{\_EP}}^{2}$ seemed to slightly increase with the COC amount, it could be concluded that the thermal behavior of the epoxy matrix was not substantially affected by the COC introduction. The systematic T_g_ increase observed in the second heating scan was due to the completion of the crosslinking of the EP matrix in the first heating stage. In the case of COC, the $T_{g_{\_COC}}^{2}$ values were slightly lower than the corresponding $T_{g_{\_COC}}^{1}$ ones. This could be due to a partial degradation of the COC structure with the applied thermal treatment. However, this effect was not dramatic, and it could be concluded that the thermal behavior of the EP and COC phases within these composites seemed to be independent of the relative composition of the constituents.

**Figure 8:**
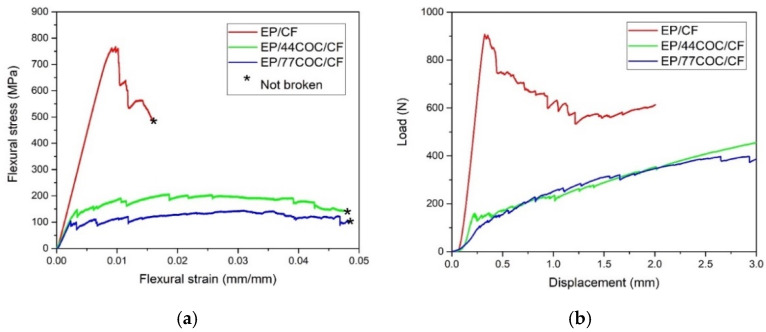
Mechanical tests on the prepared composite laminates. (**a**) Representative flexural stress–strain curves; and (**b**) load-displacement curves from short-beam shear tests.

Representative stress–strain curves of the three-point bending test and short-beam shear test on the prepared laminates are reported in Figure 8. The most important results from the tests are summarized in Table 5.

The EP/CF sample showed the typical brittle behavior of composites, in which the flexural stress exhibited a maximum, corresponding to the first breakage of the fiber layer, after which the load support started to decrease in a stepwise behavior. EP/44COC/CF and EP/77COC/CF showed a different flexural behavior, without a sudden drop of the load during the test. This indicated that the introduction of the COC layers strongly affected the mechanical behavior of the specimens, and the premature failure in these laminates was caused by a poor interlaminar adhesion between the COC layer and the matrix. The flexural modulus decreased from 64.2 GPa of EP/CF to 51.1 GPa of EP/44COC/CF, which was mainly due to the decrease in the fiber volume fraction, with the addition of COC. However, the elastic modulus of the EP/COC/CF laminates did not seem to be influenced by the thickness of the COC layers. On the other hand, the insertion of the COC layers in interlaminar position decreased drastically the flexural strength of the composite, and this effect was even more evident with thicker COC layers, probably due to an increase in porosity. This was a major drawback of these systems and would be the object of further investigation in next research activities.

Representative load-displacement curves of the SBS test are shown in Figure 8b, while the ILSS values are summarized in Table 5. The maximum load and the ILSS decreased considerably with the addition of COC, and this was more evident for the thicker COC films. The ILSS decreased from 45.9 MPa of the neat EP/CF laminate to 9.0 MPa for the EP/77COC/CF sample. This was again due to the poor adhesion between the COC films and the epoxy matrix and the increase in porosity.

### 3.2. Evaluation of the Healing Efficiency

In order to evaluate the effect of the COC addition of the electrical properties of the prepared laminates and to determine the current and voltage levels to apply to heal the samples at about 190 °C, a four-probe method was performed, and the electrical resistivity of the composites was determined. Table 6 reports the values of resistivity of the samples and the range of voltage and current applied to the samples, to activate the self-healing mechanism at the selected healing temperature (i.e., 190 °C).

Considering the standard deviation values associated with these measurements, it could be concluded that neither does the COC foil introduction nor do their thickness significantly influence the resistivity of samples. Consequently, the samples could be healed by applying similar voltage and current levels at 190 °C. This probably meant that the decrease of the CF volume fraction detected in the EP/44COC/CF and EP/77COC/CF samples, did not significantly deteriorate the conductive behavior of the laminates.

**Figure 9:**
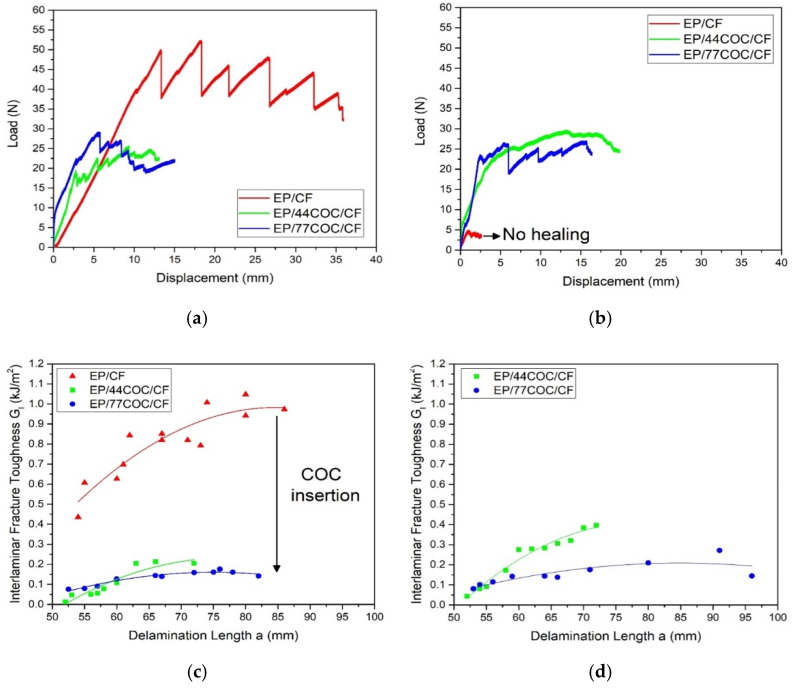
Results of mode I interlaminar fracture toughness tests. (**a**) Representative load-displacement curves of the virgin (unhealed) samples, (**b**) representative load-displacement curves of the healed samples, (**c**) representative trend of $G_{I}$ as a function of the delamination length in the unhealed samples, and (**d**) representative trend of $G_{I}$ as a function of the delamination length in the healed samples.

The fracture behavior of the prepared samples was evaluated through mode I interlaminar fracture toughness tests, and the results are displayed in Figure 9. Figure 9a shows the representative load-displacement curves of virgin (unhealed) laminates. All samples exhibit a brittle behavior, and the crack propagation occurred in correspondence of each decreasing load step. The EP/CF was able to sustain almost the double the load as compared to the other samples, which indicated that the crack propagation in the EP/44COC/CF and EP/77COC/CF specimens was faster than that in the EP/CF laminates. This was reflected in lower values of $G_{I}$, as observable in Figure 9c, which was again due to a weak interfacial adhesion between the COC and the EP. On the other hand, $G_{I}$ was not significantly affected by the thickness of the COC layers.

Figure 9b shows the representative load-displacement curves of the repaired samples. As expected, EP/CF sample did not show any healing capacity, due to the thermosetting nature of EP, while the EP/44COC/CF and EP/77COC/CF sustained loads almost equal or higher than the virgin ones. This was a clear indication that the healing treatment was successful in softening the COC layer and performing the intrinsic healing mechanism. Higher load values for the healed laminates compared to the virgin samples could be associated with the fact that the healing treatment was performed at temperatures higher than the $T_{g}$ of EP, which resulted in increased cross-linking of the EP matrix. In agreement with this result, the values of $G_{I}$ of the repaired samples (Figure 9d) were equal or higher than those of the virgin samples. This phenomenon was related to the diffusion of the COC phase in the crack zone, which showed a higher fracture toughness with respect to the epoxy matrix. The average values of the maximum load $\left(P_{max}\right)$, $G_{IC}^{VIS}$, and of the resulting healing efficiency for the produced laminates are summarized in Table 7. The neat EP/CF sample showed limited healing efficiency (less than 2%), due to the little contribution provided by the residual cross-linking or by the softening of the epoxy resin, during the healing treatment at 190 °C. On the other hand, the healed laminates containing COC could sustain a load higher than that of unhealed ones and exhibited a higher interlaminar fracture toughness, which meant that all contribution to the repair of EP/COC/CF specimens was given by the COC thermoplastic phase. A healing efficiency of 164% could be detected in the EP/44COC/CF laminate, which was higher than the healing efficiency offered by laminate with thicker COC films (100%). Therefore, thinner COC films had a higher healing potential compared to the thicker films.

Previous works of our group on the EP/COC blends [4,33] showed that the healing efficiency increased with an increase in the COC content. However, EP/44COC/CF showed a healing efficiency better than the EP/77COC/CF. All these results suggest that an increase in the thickness of the COC layers inserted into the laminates promoted an increase in the healing efficiency, but only until a threshold beyond which the efficiency started to decrease. This could be proved by producing laminates with thinner COC layers, which would be the object of future studies.

**Figure 10:**
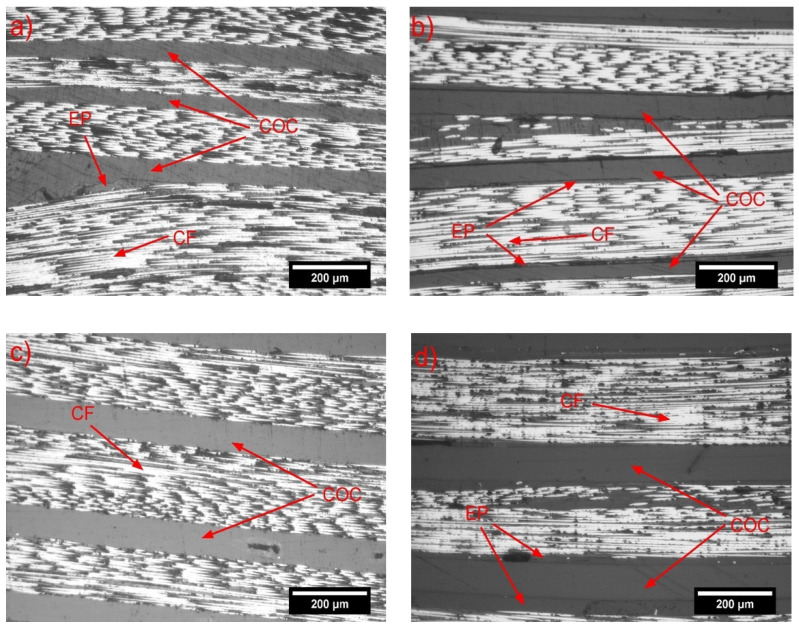
Optical microscope micrographs of the polished surfaces of unhealed and healed specimens. (**a**,**b**) EP/44COC/CF; (**c**,**d**) EP/77COC/CF. (**a**,**c**) unhealed; and (**b**,**d**) healed.

Figure 10 shows optical microscope images of the longitudinal section of the unhealed and healed samples. No particular microstructural differences due to the COC thickness could be detected in these images, and also the healing treatment did not seem to significantly affect the morphology of the prepared laminates. This meant that the observed increase of the healing capability was solely due to the flow of the softened COC within the crack front, and not because of the other microstructural effects in the composites.

## 4. Conclusions

This work reported the use of thin films of an amorphous cyclic olefin copolymer (COC) as a healing agent for epoxy/carbon (EP/CF) laminates. Thin films of two different thicknesses were created by hot pressing COC granules, and inserted in the interlaminar region of the EP/CF laminates, produced through a hand lay-up technique. The porosity was observed to increase with the introduction of the COC layers, partially because the continuous interlaminar COC films hindered a proper air removal. This effect, together with the decrease in fiber volume fraction with the COC layer thickness, contributed to degrade the mechanical properties, such as the elastic modulus, the mechanical strength, and the fracture toughness. This was a major drawback of this system, as it could limit the use of this material in structural applications. This will be the object of investigation in the next research activities. Laminates were subjected to mode I interlaminar fracture toughness tests, after which they were thermally mended by resistive heating, thanks to the Joule heating promoted by the presence of the CF layers. Healed specimens containing 44 µm and 77 µm COC layers exhibited a value of $G_{IC}$ 164% and 100% higher than that measured on virgin specimens, respectively, while the healing treatment was not effective for the neat EP/CF sample.

This work not only proved that the insertion of the COC layers was effective as a healing agent for the EP/CF laminates, but also demonstrated the efficacy of the Joule heating effect to activate the intrinsic healing mechanism. Future work will be focused on the investigation of the healing properties of thinner COC layers, and also different processing techniques (i.e., resin infusion) will be considered, to address the impairment of mechanical properties brought by the insertion of the COC interlayers.

## Figures and Tables

**Table 1:** Composition of the prepared laminates.

Composite	CF Layers	COC Layers	CF (wt.%)	EP (wt.%)	Hardener (wt.%)	COC (wt.%)	Thickness (mm)
EP/CF	14	0	78.7	16.4	4.9	0	2.66 ± 0.02
EP/44COC/CF	14	13	67.5	17.0	5.1	10.4	3.36 ± 0.02
EP/77COC/CF	14	13	62.9	14.2	4.2	18.7	3.71 ± 0.08
EP/CF	4	0	80.0	15.4	4.6	0	0.82 ± 0.01
EP/44COC/CF	4	3	70.3	15.6	4.7	9.4	1.00 ± 0.03
EP/77COC/CF	4	3	57.4	22.1	6.7	13.8	1.14 ± 0.06

**Table 2:** Weight and volume compositions of the prepared 4-layer laminates.

Composite	$\omega_{f}$ (wt.%)	$\omega_{m}$ (wt.%)	$\omega_{COC}$ (wt.%)	$\vartheta_{f}$ (vol%)	$\vartheta_{m}$ (vol%)	$\vartheta_{COC}$ (vol%)	$\vartheta_{v}$ (vol%)
EP/CF	78.7	21.3	0	68.3	28.7	0.0	3.1
EP/44COC/CF	67.5	22.1	10.4	53.0	27.0	14.4	5.6
EP/77COC/CF	62.9	18.4	18.7	47.3	21.5	24.8	6.4

$\omega_{f}$ = fiber weight fraction; $\omega_{m}$ = matrix weight fraction; $\omega_{COC}$ = COC weight fraction; $\vartheta_{f}$ = fiber volume fraction; $\vartheta_{m}$ = matrix volume fraction; $\vartheta_{COC}$ = COC volume fraction; $\vartheta_{v}$ = void volume fraction.

**Table 3:** Weight and volume compositions of the prepared 4-layer laminates.

Composite	$T_{onset}$ (°C)	$T_{d\_EP}$ (°C)	$T_{d\_COC}$ (°C)	$m_{r}$ (%)
EP/CF	336.6	366.3	-	74.2
EP/44COC/CF	341.9	370.8	477.7	67.1
EP/77COC/CF	344.3	374.5	484.3	62.3

$T_{onset}$ = onset degradation temperature; $T_{d\_EP}$ = peak temperature of the mass loss derivative signal at the degradation of EP; $T_{d\_COC}$ = peak temperature of the mass loss derivative signal at the degradation of COC; $m_{r}$ = residual mass.

**Table 4:** Results of DSC tests on the prepared laminates.

Composite	$T_{g_{\_EP}}^{1}$ (°C)	$T_{g\_COC}^{1}$ (°C)	$T_{g\_EP}^{2}$ (°C)	$T_{g\_COC}^{2}$ (°C)
EP/CF	89.08	-	90.59	-
EP/44COC/CF	88.25	82.09	92.92	72.44
EP/77COC/CF	92.28	78.78	94.45	76.63

$T_{g_{\_EP}}^{1}$, $T_{g_{\_EP}}^{2}$ = glass transition temperature of the EP phase, first and second heating scan, respectively; $T_{g_{\_COC}}^{1}$, $T_{g_{\_COC}}^{2}$ = glass transition temperature of the COC phase, first and second heating scan, respectively.

**Table 5:** Flexural properties and interlaminar shear strength (ILSS) values of the prepared laminates.

Composite	Flexural Modulus (GPa)	Flexural Strength (MPa)	ILSS (MPa)
EP/CF	64.2 ± 7.1	771.4 ± 78.3	45.9 ± 4.8
EP/44COC/CF	51.1 ± 6.5	217.2 ± 18.2	13.3 ± 0.6
EP/77COC/CF	51.4 ± 8.5	171.3 ± 28.7	9.0 ± 0.5

**Table 6:** Electrical resistivity and electrical parameters applied to heal the prepared laminates at 190 °C.

Composite	Resistivity (Ω.mm)	Voltage (V)	Current (A)
EP/CF	0.0320 ± 0.0005	4.5 ± 0.5	19.7 ± 4.9
EP/44COC/CF	0.0372 ± 0.0001	3.8 ± 0.2	18.5 ± 4.1
EP/77COC/CF	0.0378 ± 0.0002	4.1 ± 0.4	15.0 ± 4.4

**Table 7:** Results of the mode I interlaminar fracture toughness test on the virgin and healed laminates.

Composite	$P_{max}$ (N)		$G_{IC}^{VIS}$ (kJ/m^2^)		Healing Efficiency (%)
	Unhealed	Healed	Unhealed	Healed	
EP/CF	56.4 ± 3.5	16.4 ± 5.4	0.72 ± 0.07	0.01 ± 0.01	<2
EP/44COC/CF	22.3 ± 1.9	28.3 ± 1.6	0.06 ± 0.02	0.09 ± 0.01	164 ± 37
EP/77COC/CF	26.4 ± 2.5	28.6 ± 4.5	0.08 ± 0.02	0.08 ± 0.02	100 ± 12

$P_{max}$ = maximum load; $G_{IC}^{VIS}$ = interlaminar fracture toughness.
